# Microspicules in Skin Care Products: An Assessment of TikTok Reviews

**DOI:** 10.1111/jocd.71291

**Published:** 2026-11-11

**Authors:** Aaron Wang, Thu Anh Ly, Nicole Adara Case, Ruizhi Zhang, Parker Matthew Juels, Laavanya Joshi Malik, Victoria Asuquo, Robert Dellavalle, Cory A. Dunnick

**Affiliations:** ^1^ University of Colorado School of Medicine Aurora Colorado USA; ^2^ Rocky Vista University School of Osteopathic Medicine Centennial Colorado USA; ^3^ University of Minnesota Medical School Minneapolis Minnesota USA; ^4^ Morehouse School of Medicine Atlanta Georgia USA

**Keywords:** adverse‐event reporting, cosmetic safety, microspicules, promotion, reported reactions, skin care, TikTok

To the Editor,

Microspicule‐containing skincare products use microscopic, often sponge‐derived structures to disrupt the stratum corneum and enhance skin penetration of cosmetic ingredients [1, 2]. A clinical trial of one acne formulation does not establish the safety of retail microspicule products [2]. Potential concerns include irritation, skin barrier disruption, and infection [3]. We examined promotion and reported reactions in TikTok videos, then searched US Food and Drug Administration (FDA) adverse‐event records for relevant products.

Using a new TikTok account, one author searched #microspicule, #spicule, #microneedlingserum, #reedleshot, and #spiculeserum on August 13–14, 2025. Publicly viewable English‐language videos posted during the preceding 6 months were eligible; duplicates were excluded. One author logged videos in a shared spreadsheet, and coauthors screened them and manually extracted predefined variables. Commercial relationships included sponsorship, gifted products, affiliate partnerships, or store/clinic representation. Encouragement required a favorable experience and an explicit recommendation to purchase or continue use. Reaction status was coded as yes, none (an explicit statement of no reaction), or unclear (not discussed or indeterminate). Reported sensations and symptoms were not treated as clinical diagnoses. Pearson's chi‐square test compared reaction reporting by commercial status.

On September 12, 2026, we searched openFDA cosmetics, food/historical cosmetics, drug (FDA Adverse Event Reporting System [FAERS]), and device (Manufacturer and User Facility Device Experience [MAUDE]) adverse‐event records using extracted product names, brands, spelling variants, and microspicule‐related terms; drug and device searches used selected brand and class terms [4]. No event‐date restriction was applied. OpenAI Codex assisted in drafting search scripts, matching product names, and reviewing manufacturer or official‐distributor descriptions to identify microspicule‐containing products. The authors reviewed the retrieved records and product classifications. Codex also assisted with manuscript wording. Reports were counted once within each database.

Of 461 identified videos, 272 were included. Commercial relationships appeared in 191 (70.2%), and 267 (98.2%) encouraged use. Reactions were reported in 84 (30.9%); status was unclear in 181 (66.5%), and 7 (2.6%) explicitly reported none. Tingling/stinging/prickliness appeared in 68 videos (25.0%) (Table 1). Reaction reporting was not significantly associated with commercial status (*p* = 0.953). One qualifying FDA cosmetics report was retrieved for Medicube Zero Exosome Shot 7500. It described infection, rash, scar, and skin atrophy; other products were used concurrently, so causation cannot be assigned to the microspicule product. No additional qualifying reports were identified in the searched records (Table 2).

**TABLE 1:** Reaction reporting among 272 TikTok videos.

Reaction reporting	Videos, *n* (%)
None	7 (2.6)
Unclear	181 (66.5)
Yes	84 (30.9)
Reported reactions	
Tingling/stinging/prickliness	68 (25.0)
Burning or a spicy sensation	6 (2.2)
Redness	5 (1.8)
Pain	3 (1.1)
Peeling or tightness	1 (0.4)
Breakouts	1 (0.4)

*Note:* Percentages use all 272 included videos as the denominator. Unclear means reactions were not discussed or could not be determined; it does not establish tolerability. The labels reflect video coding, not clinical diagnoses.

**TABLE 2:** FDA records naming products with documented microspicule content.

Dataset	Search‐service update	Qualifying reports	Qualifying product
Cosmetics	2025‐08‐31	1	Medicube Zero Exosome Shot 7500
Food and historical cosmetics	2026‐07‐07	0	None identified
Drug events (FAERS)	2026‐07‐30	0	None identified
Device events (MAUDE)	2026‐09‐08	0	None identified

*Note:* Search performed September 12, 2026. Updates shown are openFDA search‐service dates; downloaded cosmetics and food files were last updated September 7, 2026. These dates do not establish complete coverage. Counts are distinct retrieved reports, not incidence or product registrations. Cosmetics report 25074648 (version 1) lists products used concurrently, so causation is unconfirmed. Drug and device searches used selected brand and class terms without event‐date restriction.

Nearly all sampled videos recommended use, while most did not clearly address reactions. Promotional content is also common in other dermatology TikTok samples [5]. Creator reports were not clinically verified, and the absence of a severe reaction in this sample cannot establish tolerability. Hashtag sampling and possible reaction misclassification limit generalizability. FDA report counts do not measure incidence; a limited search result cannot establish that no other reports exist. More short‐ and long‐term safety studies are needed to guide counseling about retail microspicule products.

## Author Contributions

Aaron Wang conceived the project, led project preparation, and contributed to video data extraction. Thu Anh Ly, Nicole Adara Case, Ruizhi Zhang, and Victoria Asuquo also extracted data from videos. Parker Matthew Juels contributed to manuscript preparation. Laavanya Joshi Malik contributed to statistical analysis. Robert Dellavalle and Cory A. Dunnick provided project oversight. All nine authors approved the final manuscript for submission to the *Journal of Cosmetic Dermatology*.

## Funding

The authors have nothing to report.

## Ethics Statement

This study used publicly available data; institutional review board review was not sought.

## Consent

The authors have nothing to report.

## Conflicts of Interest

Robert Dellavalle reports royalties from Wolters Kluwer for his role as an UpToDate Dermatology section editor and consulting fees from UCB related to the global burden of skin disease. He has an uncompensated relationship with the American Board of Dermatology. He is Editor‐in‐Chief of *JMIR Dermatology* and receives an editorial stipend and reimbursement of meeting expenses for this role. Cory A. Dunnick is a speaker for Pfizer Inc. All other authors report no conflicts of interest.

## Data Availability

This study analyzed publicly viewable TikTok videos (https://www.tiktok.com/) and US Food and Drug Administration adverse‐event records available through openFDA (https://open.fda.gov/). Aggregate results are reported in the article.
